# Bimodal atomic force microscopy driving the higher eigenmode in frequency-modulation mode: Implementation, advantages, disadvantages and comparison to the open-loop case

**DOI:** 10.3762/bjnano.4.20

**Published:** 2013-03-18

**Authors:** Daniel Ebeling, Santiago D Solares

**Affiliations:** 1 Department of Mechanical Engineering, University of Maryland, College Park, MD 20742, USA

**Keywords:** amplitude-modulation, atomic force microscopy, frequency-modulation, phase-locked loop, spectroscopy

## Abstract

We present an overview of the bimodal amplitude–frequency-modulation (AM-FM) imaging mode of atomic force microscopy (AFM), whereby the fundamental eigenmode is driven by using the amplitude-modulation technique (AM-AFM) while a higher eigenmode is driven by using either the constant-excitation or the constant-amplitude variant of the frequency-modulation (FM-AFM) technique. We also offer a comparison to the original bimodal AFM method, in which the higher eigenmode is driven with constant frequency and constant excitation amplitude. General as well as particular characteristics of the different driving schemes are highlighted from theoretical and experimental points of view, revealing the advantages and disadvantages of each. This study provides information and guidelines that can be useful in selecting the most appropriate operation mode to characterize different samples in the most efficient and reliable way.

## Introduction

Atomic force microscopy (AFM) emerged in the mid-1980s as a powerful tool for measuring topography and forces on micro- and nanoscale surfaces [1]. Over the years, a number of new challenges have arisen in the implementation of such characterization, which have led to highly sophisticated approaches. In 2004 Garcia and co-workers [2] reported on computational simulations of a bimodal AFM technique for the simultaneous imaging of topography and mapping of compositional contrast across the sample. Within their method the fundamental cantilever eigenmode was used to acquire the sample topography through the amplitude-modulation (AM) scheme while the second eigenmode was driven with a much smaller amplitude in open loop (OL, that is, only the first mode amplitude signal was used to control the tip–sample distance feedback loop. The second eigenmode drive signal had a constant amplitude and frequency like in standard AM-AFM, but its response was not considered in the control logic). The key advantages of this approach were (i) the ability to vary and optimize the parameters of the higher eigenmode without being restricted by the topographical acquisition control loops, and (ii) higher sensitivity of the second phase contrast to material properties in the small-amplitude regime. This method, which was later implemented experimentally [3] and studied further theoretically and computationally [4–5], gave birth to a new host of multifrequency AFM techniques, which nowadays include a wide variety of complementary methods for characterization in liquids, air and vacuum [6].

We describe here a previously introduced technique [7] similar to the original method of Garcia and co-workers (henceforth referred to as the AM-OL method), but in which the higher eigenmode is driven by using the frequency-modulation (FM, [8–9]) method, as had been previously done for vacuum operations [10–11]. Our multifrequency technique was originally introduced for ambient air operation within a trimodal scheme [12–13], in which a third active eigenmode was added to the AM-OL method. Since the dynamics of this trimodal approach are quite complex and the technique is still in the early stages of development, we focus here on a more in-depth presentation of the bimodal AM-FM method. In particular, we discuss advantages, disadvantages and differences in contrast with respect to the AM-OL approach, as well as its general applicability. The aim is to provide sufficient background to help users discern the most appropriate of these two methods for specific applications, rather than making generalizations that place one technique above the other. When appropriate we also offer brief comparisons with other imaging modes.

## Control scheme of the AM-FM mode

**Figure 1:**
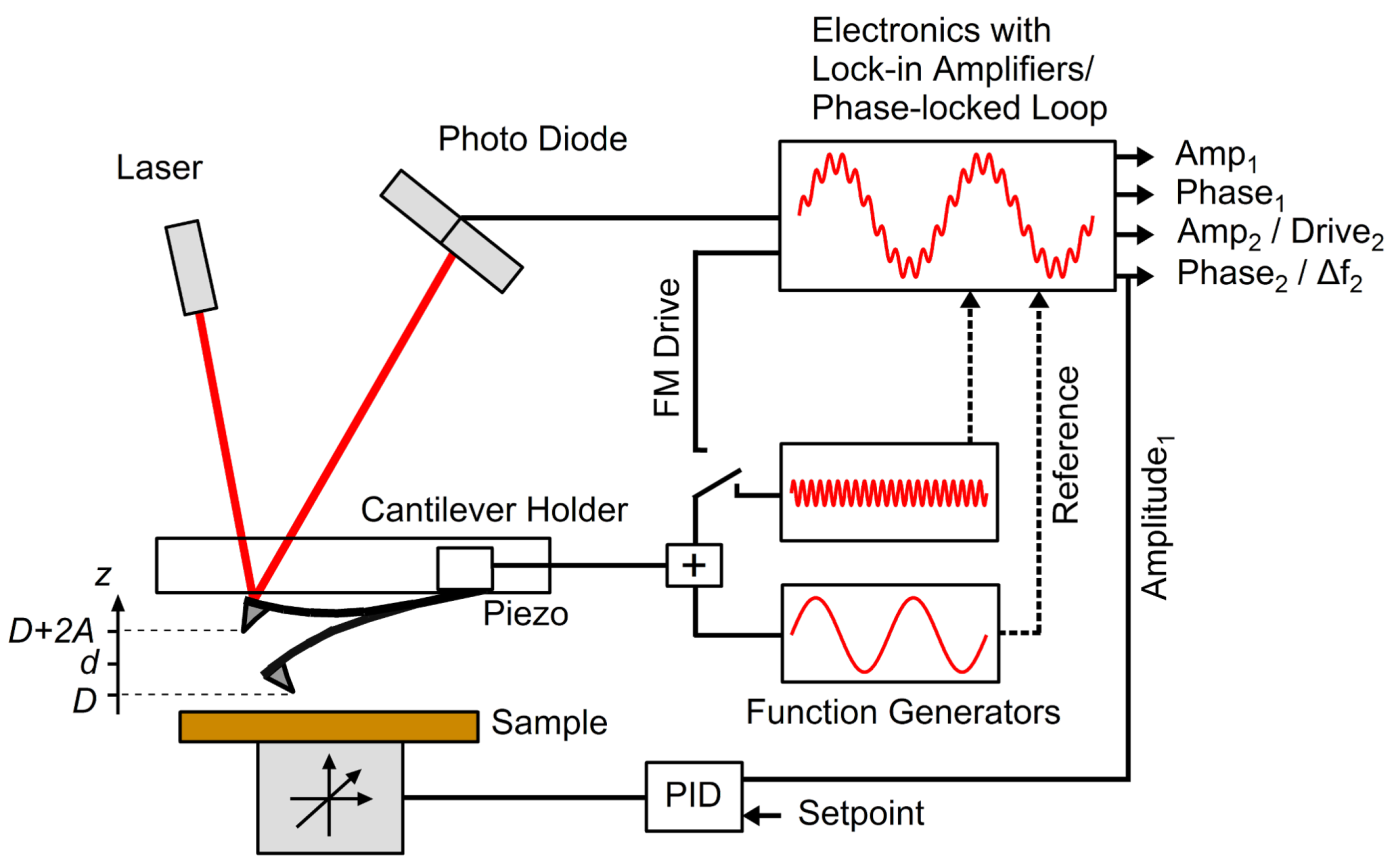
Schematic setup of our AFM operated in AM-OL or AM-FM mode. AM-OL mode can be accomplished by adding the drive signals of two function generators for exciting the cantilever beam and using two lock-in amplifiers to track the amplitudes and phase shifts at the corresponding eigenmodes. For AM-FM mode external PLL electronics were used, which provided a drive signal with a constant phase shift of 90° between excitation and response while tracking changes in the instantaneous resonant frequency.

Figure 1 shows a diagram of the experimental setup used, which consists of a commercial AFM system (MFP3D with ARC2 controller, Asylum Research Corporation, Santa Barbara, CA, USA) equipped with external phase-locked loop (PLL) electronics (PLL Pro 2, RHK Technology, Troy, MI, USA). As is customary, the oscillation of the cantilever near the sample surface was tracked with the laser beam deflection method. In the case of AM-OL operation the drive signal was generated by adding the signals of two function generators, which were set to the frequencies of the 1st and 2nd eigenmodes of the cantilever, respectively. The amplitudes and phase shifts for each eigenmode are read out by two separate lock-in amplifiers. This operation mode can be accomplished by either using the two built-in function generators and lock-in amplifiers from the MFP3D electronics or using the MFP3D electronics for the 1st eigenmode excitation only and providing the 2nd eigenmode drive signal through the RHK electronics. We decided in favor of the second option in order to be able to switch quickly between AM-OL and AM-FM operation.

In the case of AM-FM operation the 1st eigenmode of the cantilever was excited by the MFP3D controller in AM-mode, while the 2nd eigenmode was driven by the PLL in FM-mode. The PLL electronics continuously measure the instantaneous frequency of the oscillation signal and generate an excitation signal at this frequency, which is fed back to the dithering piezo (see, e.g., [14] for details) in order to keep the corresponding eigenmode always oscillating at its actual resonance frequency. The PLL can be operated in two different ways. It can either keep the drive amplitude of the excitation signal constant (this approach is often denoted as constant excitation (CE) mode [15]) or it can provide a signal with variable drive amplitude to maintain a constant oscillation amplitude of the cantilever eigenmode (accordingly denoted as constant amplitude (CA) mode [9]). The latter case is internally realized by running an additional feedback loop that controls the oscillation amplitude. Besides, since AM-OL operation can be implemented by using the MFP3D electronics alone, the setup of Figure 1 can also be employed to simultaneously excite three eigenmodes of the cantilever as described in [12–13].

## Results and Discussion

### Comparison of AM-FM and AM-OL methods

Comparisons of the AM-OL and AM-FM techniques are difficult because the two methods do not carry out identical tasks and also because there are a number of tradeoffs involved, which may or may not be advantageous depending on the particular application and level of skill of the user. We focus here on three angles of comparison: (i) the general appeal of frequency shift and relationship to phase contrast, (ii) the amplitude control capability and its implications (especially with regards to sensitivity), and (iii) complexity and stability. Additionally, we offer a brief discussion on selection criteria in terms of sample type, instrumentation availability and user skill level.

### General appeal of frequency shift and relationship to phase contrast

The first question that emerges when discussing AM-FM concerns the reasoning behind the use of FM-AFM, which has in the past been mostly reserved for vacuum operation, with a few exceptions in liquid imaging [16–17] and spectroscopy experiments in air as well as in liquid [18–21]. Historically, FM-AFM addressed the limitation brought about by the large transient times observed in classical AM-AFM, where the oscillation amplitude is used as an input signal for the tip–sample-distance feedback loop. These transient times scale as 2Q/ω_0_, with Q being the quality factor and ω_0_ the natural frequency [22]. Clearly, imaging becomes impractical when Q increases significantly (as in vacuum operations). In FM-AFM, this drawback is overcome by using the frequency shift as a feedback input, which shows an instantaneous response to variations in the tip–sample forces (on the order of the oscillation period). However, FM-AFM can also be attractive for nonvacuum operations, primarily because of its suitability for spectroscopy experiments. For CA-mode operation it can be shown that the frequency shift signal is, at a first approximation, only affected by conservative interactions while the measured drive amplitude is mainly influenced by dissipation [23–25]. However, in AM-AFM both measured signals (amplitude and phase shift) depend on both types of interactions (conservative and dissipative) [26] (note that the measured frequency shift is also *indirectly* affected by dissipation in large-amplitude intermittent-contact experiments, in that dissipative forces can limit penetration of the probe tip into the repulsive region of the tip–sample interaction potential, thus leading to lower frequency shifts [13]). The above fact complicates the reconstruction of tip–sample interactions when performing spectroscopy in AM-AFM (see [26]). Furthermore, known bistabilities from AM operation [22,27–28] do not occur in FM-AFM, ensuring smoother characterization in some cases, as well as facilitating mathematical reconstruction of the force curves and ensuring continuous acquisition of data without any jumps in the signals or cantilever response [29–30]. This is highly relevant in multifrequency operation, where one seeks to integrate imaging and spectroscopy. Finally, FM-AFM also has the potential advantage to enable real-time 3D force spectroscopy in multifrequency operation, *in the limit of small response time.* As previously simulated [31–32], if a sufficiently high eigenmode were *self-excited* while performing intermittent contact imaging with the fundamental eigenmode, such that each higher-mode oscillation remained at the instantaneous resonance frequency, one could reconstruct the tip–sample force gradient in the volume above the sample, defined by the raster scan and the oscillation amplitude of the fundamental mode. It is straightforward to carry out integration of the force gradient to calculate the forces as a function of the *xyz-*coordinates. This results in three dimensional force fields, as they are usually obtained in time-consuming volume-scanning applications [33–34]. Although the approach described in [31–32] is not yet experimentally feasible, it represents a promising theoretical limit.

**Figure 2:**
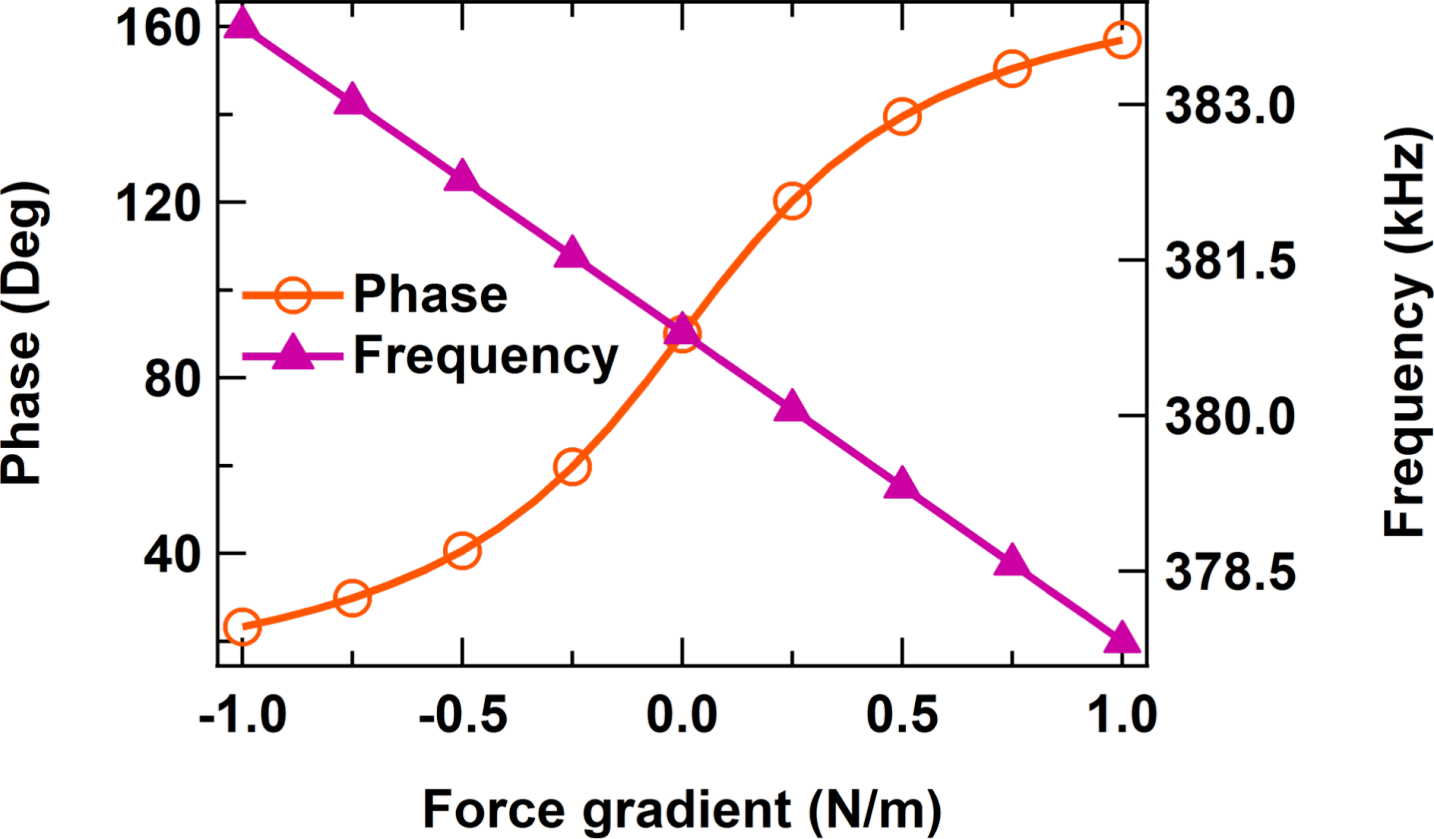
Phase and frequency shift calculated analytically for a higher eigenmode with *f*_2_ = 380.8 kHz, *k*_2_ = 64.2 N/m and Q_2_ = 450 as a function of the effective tip–sample force gradient.

The next question concerns the relationship between the frequency shift and the phase contrast, which can be easily answered by using the damped harmonic oscillator model [22]. In the absence of tip–sample dissipation, the phase of the oscillator’s response with respect to the excitation, 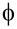, can be calculated through the expression,

[1]
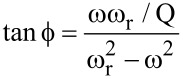


where ω and ω_r_ are the excitation and resonance angular frequencies, respectively. The angular resonance frequency is, at a first approximation, related to the *effective* force gradient experienced by the oscillator through the equation,

[2]
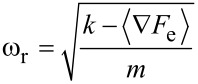


where *k* is the oscillator force constant, *F*_e_ is the external force (the tip–sample force in this case) and *m* is its effective mass, equal to 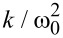. Substituting Equation 2 into Equation 1 and setting the excitation frequency to be equal to the free resonance frequency, one obtains an equation that relates the response phase to the effective force gradient, which is plotted in Figure 2 together with the resonance frequency, as a function of the effective force gradient [12–13]. The graphs show that the phase and frequency shift vary in opposite (antiparallel) directions when the magnitude of the force gradient is small [12–13]. Figure 2 also highlights one additional advantage of using the frequency shift in that its behavior remains closer to a linear response for much larger magnitudes of the external force gradient, as compared to the phase response. The nearly anti-parallel relationship between phase and frequency can be easily observed in a trimodal experiment in which one higher eigenmode is driven in OL and another one is driven in FM [12–13], or in separate bimodal experiments conducted in AM-OL or AM-FM [7].

**Figure 3:**
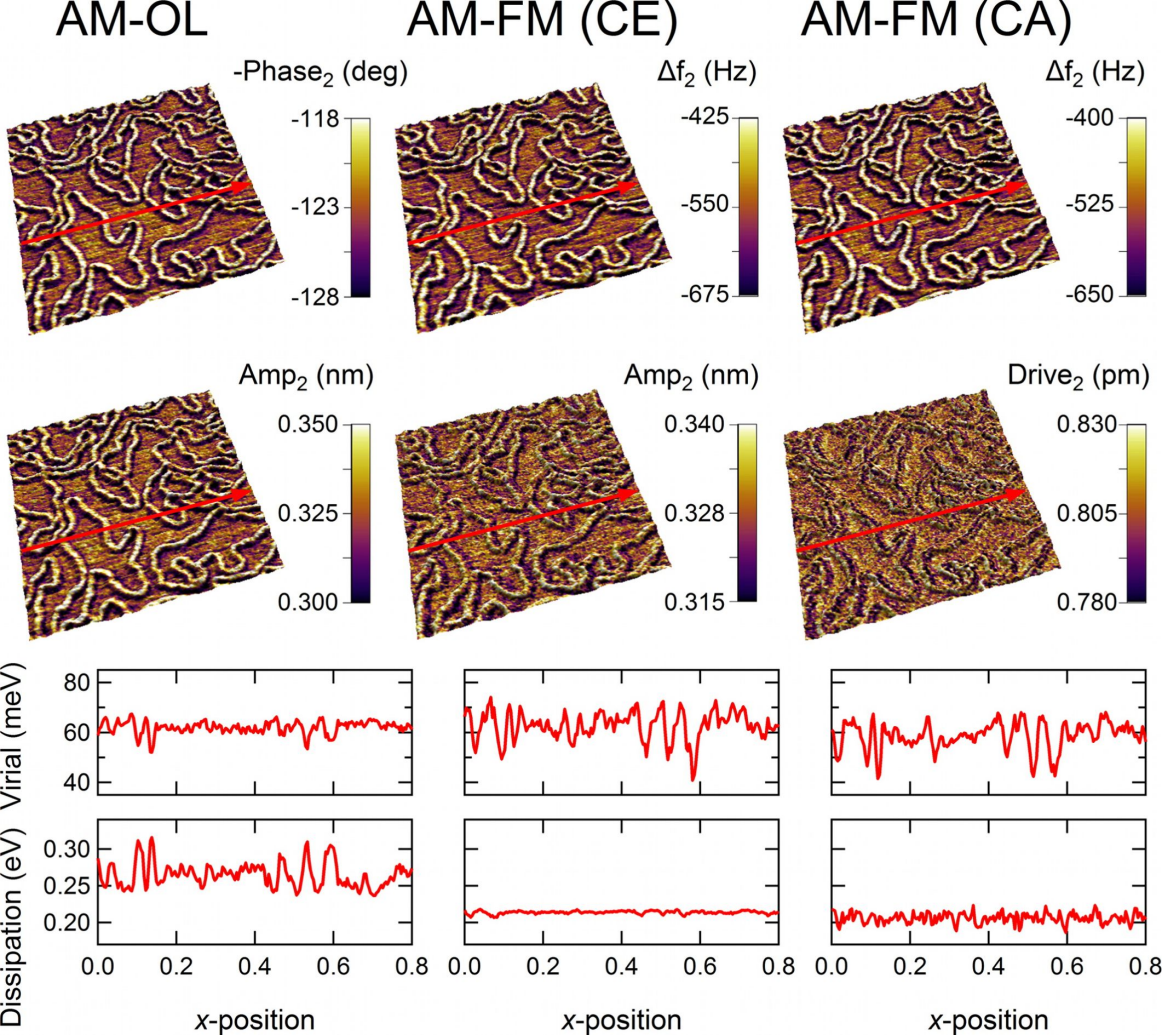
Comparison of 2nd eigenmode contrasts for different operation modes (left: AM-OL, middle: AM-FM (CE), right: AM-FM (CA). The two top rows show 3D topography images (800 × 800 nm^2^) of lambda DNA strands adsorbed onto a mica substrate. For each operation mode the topography images are overlaid by their corresponding 2nd eigenmode signal channels, which are phase shift (inverted) and oscillation amplitude (AM-OL), frequency shift and oscillation amplitude (AM-FM (CE)), or frequency shift and drive amplitude (AM-FM (CA)). In the two bottom rows the virial and energy dissipation for the scan line indicated are depicted. Parameters: rectangular silicon cantilever (PPP-NCSTAuD, Nanosensors), *f*_1_ = 92.4 kHz, *k*_1_ = 2.0 N/m, Q_1_ = 208, *f*_2_ = 586.8 kHz, *k*_2_ = 97.6 N/m, Q_2_ = 678).

Figure 3 shows a comparison of bimodal AFM measurements of DNA strands adsorbed on a mica surface obtained in three different operation modes under ambient air (sample preparation details are provided below). The images were in each case captured over nearly the same sample position in AM-OL (left), AM-FM (CE) (middle), and AM-FM (CA) (right) modes, respectively. The 3D topography images are overlaid by color-scaled images of the corresponding 2nd eigenmode channels. To demonstrate the antiparallel contrasts of phase and frequency shifts we inverted the phase image (top left image) resulting in a very similar contrast as observed for the two frequency-shift channels (top middle and right). The 2nd eigenmode oscillation amplitude (AM-OL and AM-FM (CE)) and drive amplitude (AM-FM (CA)) channels are depicted in the second row of Figure 3. For these channels a clear difference between AM-OL and AM-FM operation is observed revealing the strongest contrast in the AM-mode. To be able to compare the contrast in the different modes more quantitatively, we converted the measured phase/frequency shifts and amplitudes into virial and energy dissipation, which are the time averages of the conservative and dissipative tip–sample interactions, respectively (see, e.g., equations (1a) and (1b) in [7] and references therein. Here we converted the dissipated power into energy dissipation per oscillation cycle by multiplying the power by the period length). These quantities are depicted in the two bottom rows of Figure 3 for one scan line. For the specific sample system and the actual imaging parameters used, both virial and dissipation channels reveal significant differences between AM and FM operation. While in AM mode the image contrast shows up in both channels almost equally, the FM data show highly diminished contrast in the dissipation channel but in return a slightly increased contrast for the virial.

The cause for this behavior lies in the complex inherent characteristics of the different excitation mechanisms. For guaranteeing better comparability of the data from diverse operation modes the average 2nd eigenmode oscillation amplitudes were adjusted to the same value (approx. 0.33 nm) during the experiments. To accomplish the same engaged amplitude, three different free amplitudes (that is, amplitudes without tip–sample interaction) had to be used, which were 0.75 nm (AM), 0.58 nm (CE) and 0.33 nm (CA) [35]. In light of this fact it becomes clear that a direct comparison of the various excitation schemes is difficult and has to be conducted very carefully, especially for highly dissipative sample systems. The comparison of OL, CE and CA presented in [7] seems more straightforward than for the results presented here, because in those experiments the engaged amplitude of the spectroscopic eigenmode does not differ significantly from the free oscillation amplitude for OL and CE. This is primarily a consequence of using the 3rd cantilever eigenmode in that study instead of the 2nd eigenmode used here (the 3rd eigenmode is significantly stiffer than the 2nd, and is thus influenced by the tip–sample forces to a lesser degree). However, this is not generally the case. Instead, the ratio of engaged to free amplitude can be small and differ between OL and CE throughout the sample.

**Figure 4:**
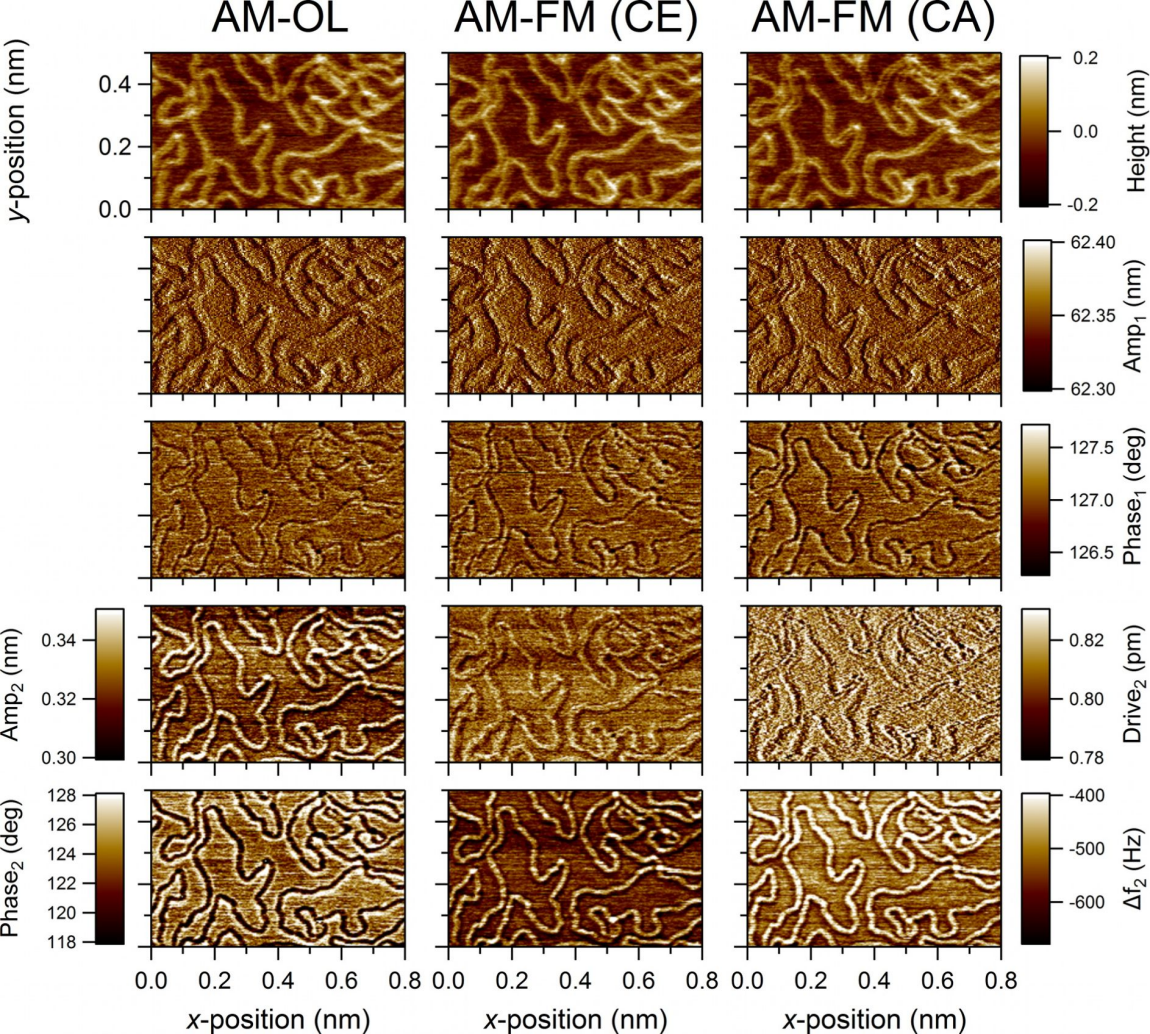
500 × 800 nm^2^ sized images of DNA/mica samples in three different multifrequency AFM modes (AM-OL, AM-FM (CE), AM-FM (CA)). Data channels in rows from top to bottom show: height, 1st amplitude, 1st phase shift, 2nd amplitude (oscillation or drive), 2nd phase or frequency shift (see Figure 3 for parameters).

In Figure 4 all data channels that were captured during bimodal operation are presented for the different operation modes. Shown are 500 × 800 nm^2^ 2D images of the same sample location as in Figure 3. The rows from top to bottom show the height, 1st amplitude, 1st phase, 2nd (oscillation or drive) amplitude, and 2nd phase or frequency-shift channels. Whenever possible, images in the same rows were scaled to the same ranges. Here we observed an increased contrast for the 2nd eigenmode signals in comparison to the corresponding 1st mode signals in agreement with previous results reported in the literature (see, e.g., [3,10,36–38]). It is interesting to note that the 2nd mode phase and frequency-shift images reveal a significantly improved lateral resolution in comparison to the height channels. Especially at locations where DNA strands lay close to each other the height contrast becomes ambiguous, not allowing for an identification of single strands. However, in the 2nd phase and frequency-shift channels the borderlines of the single DNA strands are clearly visible. Furthermore, comparing the 1st and 2nd phase channels in AM-OL mode it becomes apparent that the observed image contrast in the 2nd eigenmode channel is about one order of magnitude higher than in the 1st mode channel for the chosen parameters. On closer examination of the 2nd eigenmode channels in Figure 4 one notices some asymmetry in the image contrast around the DNA strands in all three operation modes. We checked whether this effect is influenced by the scanning direction but this was not the case. We believe that this asymmetry is likely to be related to the shape of the tip apex.

### Amplitude control capability and its implications

**Figure 5:**
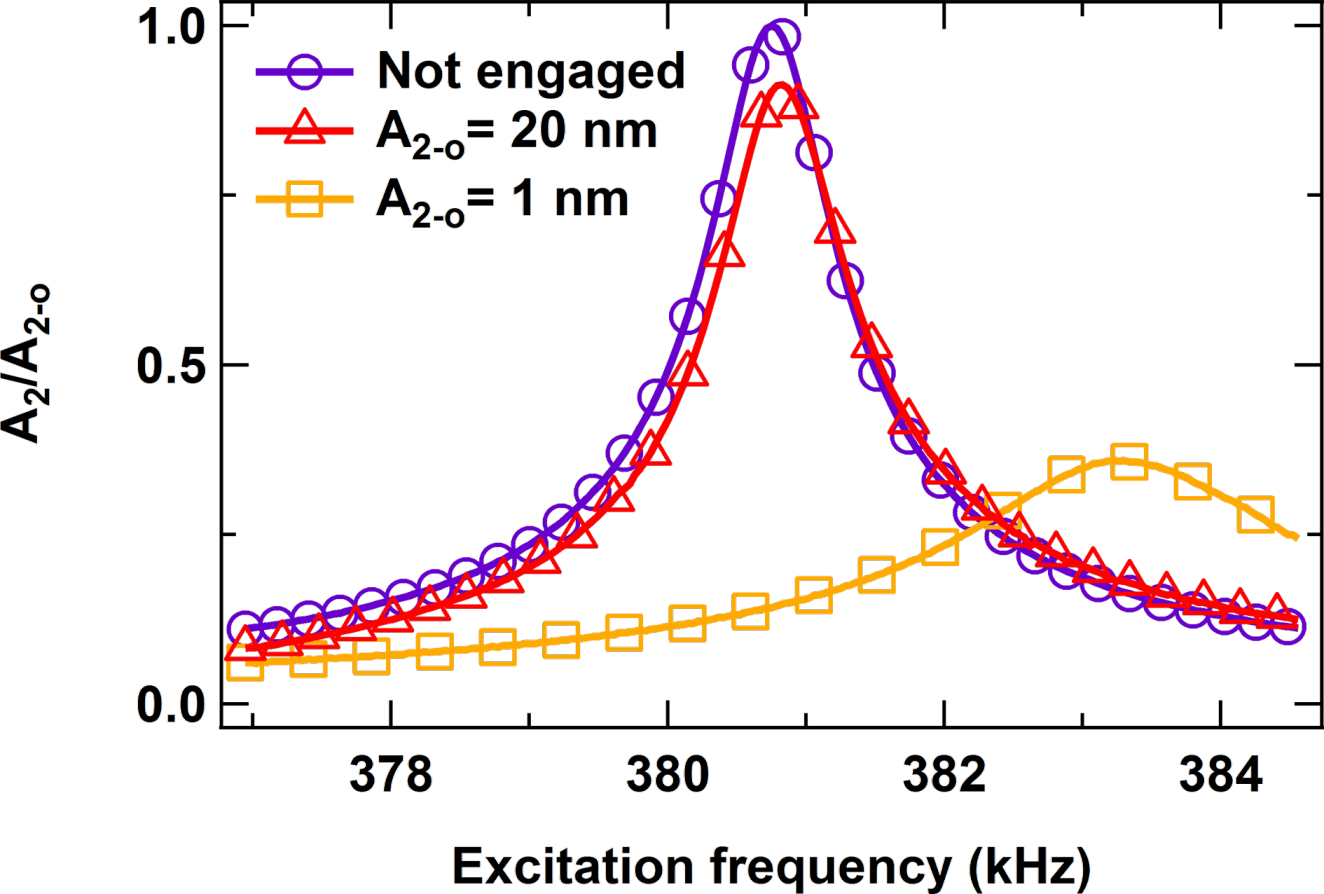
Simulation of the change in frequency response for the second eigenmode of a cantilever with fundamental eigenfrequency of 60.8 kHz (Q ≈ 150), second eigenfrequency of 380.8 kHz (Q ≈ 450), and fundamental force constant 1.6 N/m as a function of the second eigenmode’s free oscillation amplitude. The fundamental free oscillation amplitude and amplitude setpoint are 100 nm and 70%, respectively. The sample is a soft dissipative polymer [13]. The graph illustrates the increased sensitivity of the second eigenmode as its free amplitude drops from 20 nm to 1 nm. In the former case the frequency shift and decrease in response amplitude are small compared to the free response. In the latter case, the eigenmode is much more sensitive, leading to a significantly larger frequency shift and much smaller oscillation amplitude.

**Figure 6:**
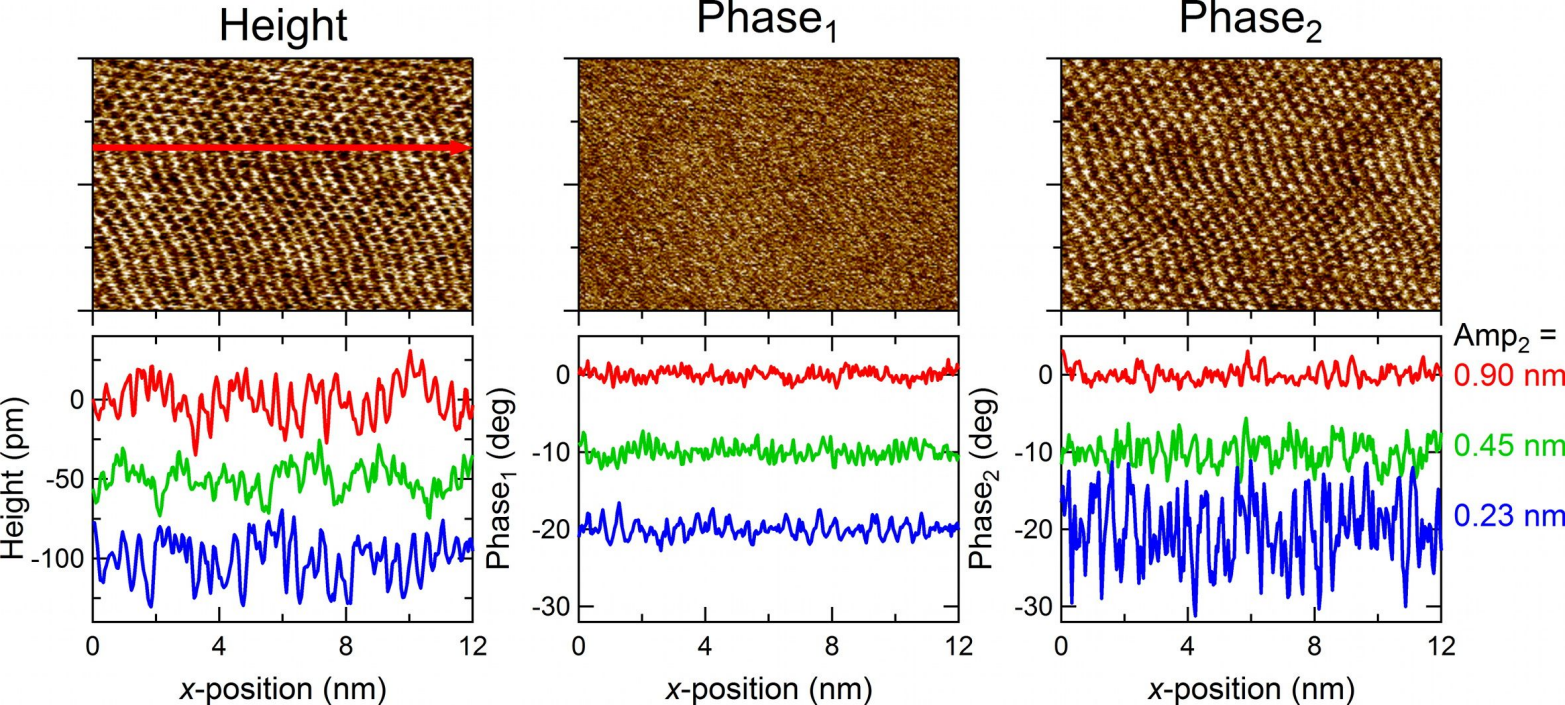
Top row: 8 × 12 nm^2^ height, 1st phase shift, and 2nd phase shift images of a mica surface imaged in ultrapure water in AM-OL mode. Bottom row: single scan lines for three different 2nd mode amplitudes (0.90, 0.45, 0.23 nm). Parameters: rectangular silicon cantilever (PPP-NCSTAuD, Nanosensors), 1st free amplitude = 1.5 nm, amplitude setpoint = 0.75 nm.

An important advantage of the AM-FM scheme with respect to the AM-OL method is that the spectroscopy eigenmode operates at a fixed phase of 90 degrees, corresponding to the natural frequency, where the cantilever is generally most sensitive to external forces and thus permits characterization with gentler impacts. Although the maximum amplitude (peak in the Lorentzian response) does not occur exactly at the natural frequency due to the influence of damping, which can be more significant when characterizing highly dissipative samples, the natural frequency is a well-defined condition, which allows the relatively easy implementation of amplitude control. That is, one can control the response amplitude by adjusting the drive amplitude, using a simple control loop, such that FM-AFM can be carried out either by using the constant excitation (CE) mode [15] or the constant amplitude (CA) mode [9]. In the former case the controller simply adjusts the drive frequency proportionally, letting the response amplitude vary when dissipation is present, while in the latter case the drive is also adjusted to keep the response amplitude constant. Due to the Lorentzian behavior of each eigenmode, which leads to amplitude and phase responses that depend *nonlinearly* on the ratio of the excitation frequency to the *instantaneous* resonance frequency (that is, the resonance frequency under the influence of tip–sample forces), implementing a constant amplitude scheme when the phase is not locked is not as simple as rescaling the drive amplitude, although it is possible and has been demonstrated experimentally [39–41]. (Although our analysis does not address the use of phase modulation in multifrequency operations, it is worth mentioning that the phase-modulation approach has been proposed to be potentially very sensitive and fast due to relying on a phase detector, which has a faster response than a PLL and is not subject to loss of oscillation as in the case when a PLL unlocks [40]). The ability to keep the amplitude constant has direct implications on whether all regions of the sample are characterized with the same probe sensitivity. This can be understood by making dimensionless the equation of motion of a damped harmonic oscillator [22,42–43]:

[3]
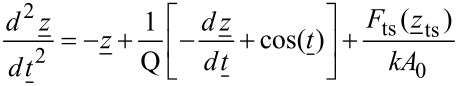


where *A*_0_ is the free oscillation amplitude, *z* = *z*(*t*)/*A*_0_ is the dimensionless tip position with respect to the cantilever base position, *z*_ts_ = *z*_ts_/*A*_0_ is the dimensionless tip–sample distance (*z*_ts_ = *z* + *z*_eq_, where *z*_eq_ is the equilibrium tip position with respect to the sample surface), *t* = ω_0_*t* is the dimensionless time, *k* is the cantilever force constant (stiffness) and *F*_ts_ is the tip–sample interaction force. We have also used the approximation *A* ≈ *A*_0_ = *F*_0_Q/*k* [22], where *F*_0_ is the amplitude of the inertial excitation force, and have grouped the damping and excitation terms together in brackets with the coefficient 1/Q*.* It can be inferred from the last term on the right hand side of this equation that the tip–sample forces are normalized by the product of the force constant times the free oscillation amplitude, such that the external force term becomes more or less relevant to the dynamics when the product *kA*_0_ becomes smaller or larger, respectively. Since the cantilever becomes more sensitive to external forces when this term becomes more dominant, sensitivity increases with decreasing amplitude. Thus, unless the amplitude is kept constant across the surface, regions of the sample where dissipation is high will lead to smaller amplitudes and, thus, will be characterized with higher cantilever sensitivity, leading to unequal treatment of all regions [7]. Clearly this is not a concern for samples that exhibit low dissipation or for cases where the user is able to set the amplitude to a value that is large enough to prevent the external force term from becoming dominant (see the trace for a free second eigenmode amplitude of *A*_2-0_ = 20 nm in Figure 5, for which the influence of the external forces is small in comparison to the trace for *A*_2-0_ = 1 nm), although the use of larger amplitudes leads to lower sensitivity and reduced material contrast, so it is not necessarily desirable. Similarly, there may be situations where the user is interested in increasing the sensitivity of the instrument across the entire sample. For example, in the detection of atomic-scale features in liquid environments, the use of increasingly smaller oscillation amplitudes leads to gradually increased dominance of the tip–sample forces in Equation 3, allowing the user to find optimum conditions that balance cantilever sensitivity with the ability of the instrumentation to detect changes in the signals. Figure 6 shows an example of this approach for imaging the mica–water interface using AM-OL. The top row in Figure 6 depicts high-resolution topography as well as 1st and 2nd eigenmode phase-shift images of a mica surface imaged in ultrapure water. The images reveal clearly the hexagonal structure of the mica surface and, at certain positions, single atomic/molecular adsorbates/defects. In the bottom row single scan lines for three different 2nd mode amplitudes are compared with each other. The red scan lines were taken from the images in the top row (position indicated by red arrow). The 2nd mode phase-shift scan lines reveal a significant increase in contrast for decreasing 2nd mode amplitudes. Here it is important to realize that Equation 3 is valid for any harmonic oscillator, so the ability to tune its sensitivity is not limited to bimodal operations. In fact, we have also observed similar trends as in Figure 6 when performing the characterization using a single eigenmode [38].

### Complexity and stability

As expected from a more sophisticated method having more “knobs,” AM-FM offers useful advantages with respect to AM-OL. However, this also comes at a price, which may or may not be worth paying. Specifically, the controls on the spectroscopy eigenmode are significantly more complex and the instrumentation more expensive. In AM-OL this eigenmode is driven at constant amplitude and frequency but not controlled. In this case “simple” lock-in amplifiers are sufficient to rapidly measure oscillation amplitudes and phase shifts at each eigenmode of the cantilever. In contrast, the CE version of AM-FM requires either a phase-locked-loop (PLL) or a self-excitation (phase-shift-based) loop to keep the phase locked at 90 degrees (so far we have observed that self-excitation loops are less stable in tapping-mode experiments using the same setup as in Figure 1, as it is very easy for the system to lose resonance during the tip–sample impact, where the instantaneous resonance frequency, and thus the length and required phase shift of each successive oscillation, changes rapidly). The CA version of AM-FM requires an additional control loop to keep the amplitude constant. This added complexity is not necessary for many samples of interest, for which AM-OL is sufficient. Furthermore, due to its robustness, AM-OL can be more advantageous when characterizing samples with properties (dissipation, stiffness or adhesive forces) that exhibit sharp variations across a wide range. Such variations pose challenges in AM-FM because the PLL control loops need to be tuned to the expected type of external forces, such that when these forces vary considerably, the operation may be detuned (and thus, less responsive or unstable at least to some degree) part of the time. If this leads to loss of lock in the PLL the user will be unable to obtain an image, in contrast to AM-OL, which will still produce an image. As an example consider the influence of dissipative forces, which can significantly lower the effective quality factor of the spectroscopy eigenmode. As discussed in [13] and illustrated by the 1 nm trace in Figure 5 (see orange trace for *A*_2-0_ = 1 nm, which shows a drastic drop in the eigenmode’s quality factor with respect to the red trace for *A*_2-0_ = 20 nm), it is not unlikely that the effective quality factor of a higher eigenmode can become lower than the free quality factor of the fundamental eigenmode when dissipation is significant or small amplitudes are selected (as stated above, one may do this in order to measure with higher sensitivity). If the user tunes the PLL for free response, which exhibits a sharper Lorentzian curve than when tip–sample dissipative forces are present, it will necessarily be detuned upon engaging the sample with the cantilever, leading to a sluggish response.

On the other hand, the AM mode is known to be frequently accompanied by bistabilities [22,27–28] which may, depending on the sample and cantilever properties, significantly impair its imaging and spectroscopy capabilities. These bistabilities result from the existence of different oscillation states of the cantilever in AM-mode. Under certain imaging conditions it can happen that the oscillation jumps back and forth between those states, making proper tracking of the sample surface impossible. During spectroscopy measurements this phenomenon leads to discontinuities in the corresponding amplitude- and phase-versus-distance curves. Usually, these bistability issues do not occur in FM operation, since in this case, the cantilever is always driven at its actual resonance frequency [29].

### Selection criteria

As can be gathered from the previous paragraphs, the different excitation mechanisms have their advantages and disadvantages both in terms of complexity and ease of data interpretation. It is well-known from single-mode operation that AM-mode is quite robust since it neither involves “locking” the phase with the PLL electronics nor setting up parameters (e.g., gains) to tune the system prior to characterization. On the other hand, it can be subject to bistabilities that may in some cases impair imaging as well as spectroscopy capabilities. For FM-operation, PLL or self-oscillation electronics are needed, which are more expensive and complex to use. Properly “locking” the phase and additional feedback loops for keeping the amplitude constant can be handicaps of this mode, especially when the user is not sufficiently experienced. However, the benefits are operation without bistabilities, straightforward way of separating and/or calculating the conservative and dissipative tip–sample interactions, and the ability to image with constant amplitude and thus uniform sensitivity. The advantages and disadvantages of each mode are similar in bimodal operation, although their relative importance can vary. For example, bistability issues are not as detrimental as in single-mode operation. Direct comparisons are more difficult in multifrequency operation because, although some correspondence can be established between the contrast obtained from the different methods, there is not yet a *direct quantitative* relationship between them, especially when dissipation is present. The decision on which imaging mode to use should in general depend on the type of application and the purpose of the experiment. If, for example, topographical imaging of the sample surface with a general idea of the compositional contrast is the main objective (for example in the identification of two components in a sample), then it may be advantageous to profit from the robustness and simplicity of AM-OL mode. However, if inhomogeneous samples are involved, which contain wide variations in elastic and dissipative properties, and more “quantitative” data is sought, it may be advantageous to use the AM-FM scheme, which can guarantee constant sensitivity across the sample, even if the characterization is more complex and time-consuming (AM-OL can also be used with uniform sensitivity when the sample can be characterized with small variations in the engaged amplitude of the higher eigenmode with respect to the free response).

## Conclusion

We have presented a theoretical and experimental comparison of three different bimodal AFM operation schemes, namely AM-OL, AM-FM (CE), and AM-FM (CA), which differ in the type of control scheme used to drive the higher eigenmode (open loop, constant-excitation frequency-modulation, and constant-amplitude frequency-modulation, respectively). The corresponding higher eigenmode channels exhibit clear differences in each case, which are closely related to dynamics and complexity of their corresponding driving mechanisms. In general, the AM-OL operation mode comes with ease of use, low requirements for special equipment, and robustness of operation. On the other hand, AM-FM is advantageous in enabling spectroscopy without “jumps”, straightforward reconstruction of the tip–sample interaction force and the ability to operate with constant response amplitude, thus ensuring uniform sensitivity across the sample. Although generalized comparisons are not possible and it remains a challenge for the experimentalist to select the operation mode that best fits the actual application, we provide guidelines and physical insight that are helpful in making this selection.

## Experimental

Lambda DNA (New England Biolabs) was obtained in buffer solution at a concentration of 500 μg/mL. This was diluted by adding ultrapure water (Sigma) and 1M CaCl_2_ solution (Sigma) to a final concentration of 10 μg/mL DNA in 10 mM CaCl_2_. Small amounts of this solution were stored at −20 °C for later use. Directly before imaging, 20 μL of the DNA in CaCl_2_ solution was dropped onto a freshly cleaved muscovite mica (Ted Pella) surface. After waiting for approximately one minute the samples were first blown dry by air before being rinsed with ultrapure water and subsequently blown dry again. After this the samples were imaged in ambient air. For the high-resolution experiments (Figure 6) the mica samples were freshly cleaved and imaged in a droplet of ultrapure water. In this case the cantilever and its holder were rinsed by isopropanol, ethanol and water prior to imaging.
